# Ultrasound-guided lumbar medial branch blocks and intra-articular facet joint injections: a systematic review and meta-analysis

**DOI:** 10.1097/PR9.0000000000001008

**Published:** 2022-05-16

**Authors:** Zachary M. Ashmore, Michael M. Bies, James B. Meiling, Rajat N. Moman, Leslie C. Hassett, Christine L. Hunt, Steven P. Cohen, W. Michael Hooten

**Affiliations:** a Department of Physical Medicine and Rehabilitation, Mayo Clinic Graduate School of Medicine, Rochester, MN, USA; b Department of Anesthesiology, Division of Pain Medicine, Washington University School of Medicine, St. Louis, MO, USA; c Mayo Clinic Libraries, Mayo Clinic, Rochester, MN, USA; d Department of Pain Medicine, Mayo Clinic Florida, Jacksonville, FL, USA; e Department of Anesthesiology and Critical Care Medicine, Johns Hopkins School of Medicine, Baltimore, MD, USA; f Department of Anesthesiology and Perioperative Medicine, Mayo Clinic, Rochester, MN, USA

**Keywords:** Ultrasound, Medial branch block, Facet joint injection, Systematic review, Meta-analysis

## Abstract

There is great interest in expanding the use of ultrasound (US), but new challenges exist with its application to lumbar facet–targeted procedures. The primary aim of this systematic review and meta-analysis was to determine the risk of incorrect needle placement associated with US–guided lumbar medial branch blocks (MBB) and facet joint injections (FJI) as confirmed by fluoroscopy or computerized tomography (CT). An a priori protocol was registered, and a database search was conducted. Inclusion criteria included all study types. Risk of bias was assessed using the Cochrane risk of bias tool for randomized controlled trials and the National Heart, Lung, and Blood tool for assessing risk bias for observational cohort studies. Pooled analysis of the risk difference (RD) of incorrect needle placement was calculated. Pooled analysis of 7 studies demonstrated an 11% RD (*P* < 0.0009) of incorrect needle placement for US-guided MBB confirmed using fluoroscopy with and without contrast. Pooled analysis of 3 studies demonstrated a 13% RD (*P* < 0.0001) of incorrect needle placement for US-guided FJI confirmed using CT. The time to complete a single-level MBB ranged from 2.6 to 5.0 minutes. The certainty of evidence was low to very low. Ultrasound-guided lumbar MBB and FJI are associated with a significant risk of incorrect needle placement when confirmed by fluoroscopy or CT. The technical limitations of US and individual patient factors could contribute to the risk of incorrect needle placement.

## Abstract 2

Supplemental Digital Content is Available in the Text.

In this systematic review and meta-analysis, ultrasound-guided lumbar medial branch blocks and facet joint injections were associated with significant risk of incorrect needle placement.

## 1. Introduction

Fluoroscopy is the most widely used imaging modality for performing lumbar medial branch nerve blocks (MBB) and facet joint injections (FJI).^5,7,35^ Current Procedural Terminology codes for ultrasound-guided paravertebral injections (0213T- 0218T) are considered investigational and experimental, and American Society of Interventional Pain Physicians guidelines mandate the use of fluoroscopy or computed tomography (CT) for facet interventions.^29^ However, there has been an effort to increase the use of ultrasound (US) for spine procedures, including sacroiliac joint injections, epidural steroid injections, MBB, and FJI.^22,23^ Proposed benefits of US include lower cost and avoidance of radiation exposure for patients and medical personnel.^4,26^ Although there is great interest in expanding the use of US, there are new challenges with its application to lumbar facet–targeted procedures including increased tissue depth in the lumbar region.^26^ The technological limitations of US combined with the tissue depth of lumbar facets may affect the accuracy of needle placement. This is critically important when facet-targeted procedures are used for diagnostic purposes.

The use of US to perform lumbar MBB and FJI and the associated risk of incorrect needle placement have not been previously summarized. The primary aim of this systematic review and meta-analysis was to determine the risk of incorrect needle placement associated with US-guided lumbar MBB and FJI as confirmed by fluoroscopy or CT. Secondary objectives include summarizing (1) the techniques used to perform US-guided lumbar MBB and FJI, (2) procedure time for performing US-guided lumbar MBB and FJI, and (3) complications.

## 2. Methods

### 2.1. Search strategy

This study was performed according to the Preferred Reporting Items for Systematic Reviews and Meta-Analyses guidelines,^31^ and the study protocol was registered at the International Prospective Register of Systematic Reviews (PROSPERO) (CRD42020172717) in April 2020.^2^ A comprehensive search of databases was conducted from inception to February 1, 2021, and there were no language restrictions. The databases included Ovid MEDLINE(R) and Epub Ahead of Print, In-Process & Other Nonindexed Citations and Daily, Ovid Embase, Ovid Cochrane Central Register of Controlled Trials, Ovid Cochrane Database of Systematic Reviews, and Scopus.

The search strategy was conducted by an experienced librarian with input from the principal investigator. Controlled vocabulary supplemented with keywords was used to search for studies describing US-guided MBB and FJI for low back pain. The detailed strategy listing all search terms used and how they are combined is available in the supplemental materials document (available at http://links.lww.com/PR9/A160).

### 2.2. Study selection process

Study inclusion criteria included (1) evaluation of US-guided lumbar MBB and FJI, (2) all study designs including conference proceedings and abstracts, and (3) outcomes assessing feasibility, diagnosis, prognosis, or safety. Exclusion criteria included (1) human cadaver or animal studies.

In the first review phase, 2 pairs of reviewers independently screened all titles and abstracts identified by the search strategy. In the second phase, the 2 pairs of reviewers independently screened the full text of all studies and inclusion and exclusion criteria were applied. Any reviewer disagreements were resolved by a third party.

### 2.3. Data extraction

Data were extracted by 4 independent reviewers using a templated electronic database. Based on the a priori protocol, abstracted data included the year of publication; number of participants; type of intervention; imaging technique used to perform the intervention; and outcomes assessing feasibility, diagnosis, prognosis, or safety. The corresponding authors of selected studies were contacted if missing or incomplete data were reported.

### 2.4. Risk of bias assessment

Risk of bias was assessed using the Cochrane risk of bias tool for randomized controlled trials (RoB2).^49^ The National Heart, Lung, and Blood tools for assessing risk of bias were used for case series and observational cohort studies with and without controls.^33^

### 2.5. Grading of evidence

The various outcomes assessed in this review were evaluated according to the Grading of Recommendations, Assessment, Development, and Evaluation (GRADE) approach.^18,48^ Domains of evaluation included risk of bias, imprecision, inconsistency, indirectness, and publication bias.

### 2.6. Evidence synthesis

For each study, the number of needles placed by US guidance for MBB or FJI was recorded and the number of needles confirmed by fluoroscopy or CT to be correctly placed by US guidance was also recorded. Using the inverse variance method, the risk difference of US-guided needle placement as confirmed by fluoroscopy or CT was pooled across all studies using a random effects model. Heterogeneity was expressed using the *I*^2^ statistic, and results were reported with 95% confidence intervals (95% CI). All statistical analyses were performed using RevMan (Reviewer Manager, version 5.3.5; the Cochran Collaboration, Copenhagen, Denmark).

## 3. Results

### 3.1. Characteristics of included studies

**Figure 1.**
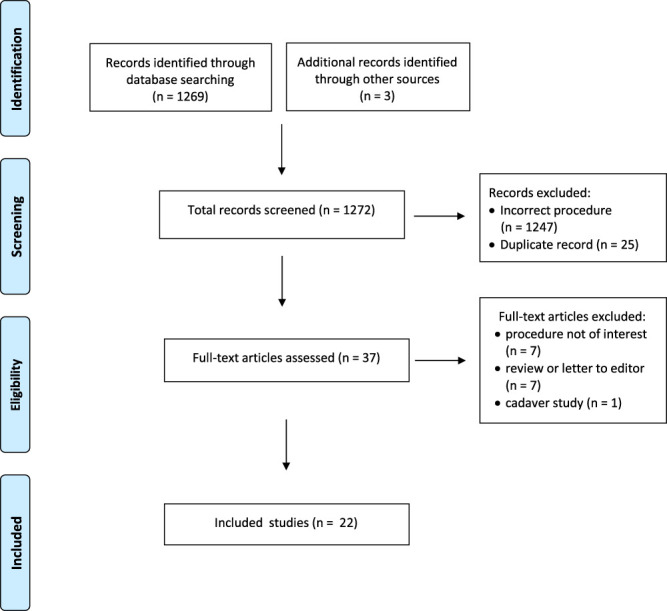
Preferred reporting items for systematic reviews and meta-analyses flowchart of study selection process.

A flow diagram of the study selection process is depicted in Figure 1. A total of 22 studies met the inclusion and exclusion criteria (Table 1).^3,9,13,14,16,17,19–21,24,25,32,37,40,41,43,47,54,55^ Study designs included 6 randomized controlled trials (RCTs),^16,19,25,51,54,55^ 1 controlled cohort study,^43^ 9 cohort studies,^3,13,14,21,24,28,32,37,47^ 1 retrospective comparative study,^20^ 3 case series,^9,17,40^ and 2 case reports.^6,41^ Four studies were conference proceedings or abstracts.^3,9,32,47^

**Table 1:** Study characteristics.

Author	Study design	No. of patients	No. US-guided blocks	Injectate	Technique	Levels blocked (number of blocks)	Confirmation method
Medial branch block							
Batalov^3^ 2013	Single-arm cohort	35	176	1 mL 0.25% bupivacaine and 5 mg methylprednisolone	US-guided “facet nerve block,” technique not specified	L2–L5 spinal levels; 17 unilateral, 18 bilateral	None
Chen^6^ 2020	Case report	1	2	0.25 mL, content not described	Transverse view to determine target (junction of SAP and superior border of TP), lateral to medial in-plane injection, longitudinal view to confirm location	L2 MBB and L3 MBB	None
Etheridge^14^ 2020	Single-arm cohort	115	100 (15 patients excluded due to inability to visualize target)	0.5 mL 0.75% bupivacaine	Longitudinal view to determine level, transverse view to locate L4 MBB target (junction of the cephalad TP and SAP), lateral to medial in-plane injection; subsequent redirection of needle medially and caudally for L5 MBB while tracking progress in a sagittal view	L4 MBB (100), L5 MBB (100); all unilateral	Fluoroscopic needle position and contrast to validate position of L5 MBB only
Greher^17^ 2004	Case series	5	28	1 mL 0.25% bupivacaine	Longitudinal view to determine level, transverse view to determine target (junction of the cephalad TP and SAP), in-plane injection, lateral to medial, verification with longitudinal view	L2 MBB (8), L3 MBB (10), L4 MBB (10); all bilateral	Fluoroscopic needle position
Han^20^ 2017	Retrospective comparative	146 (US group: 68, FL group: 78)	94	0.5 mL 1% lidocaine and 2.5 mg dexamethasone	Longitudinal scan to determine level, transverse view to determine target (junction of cephalad TP and SAP and junction of SAP and sacral ala); L5 MBB occasionally performed in out-of-plane fashion if sacral ala obstructed field of view	L3 MBB, L4 MBB, L5 MBB; number at each level not specified, number of unilateral and bilateral cases not specified	None
Hashemi^21^ 2017	Single-arm cohort	30	89	1 mL 1% lidocaine and 40 mg triamcinolone	Longitudinal view to determine level, transverse view to determine target (junction of the cephalad TP and SAP), lateral to medial in-plane injection	L3 MBB (30), L4 MBB (31), L5 MBB (28); number of unilateral and bilateral cases not specified	Fluoroscopic needle position
Jung^24^ 2012	Single-arm cohort	50	95	1 mL 2% lidocaine and 40 mg triamcinolone	Longitudinal view to determine level, transverse view to locate target (junction superior TP and SAP), lateral to medial in-plane injection	T12 MBB (1), L1 MBB (1), L2 MBB (3), L3 MBB (35), L4 MBB (48), L5 MBB (7); number of unilateral and bilateral cases not specified	Fluoroscopic needle position and contrast
Moon^32^ 2013	Single-arm cohort	27	27 patients, total number of blocks not reported	0.5% lidocaine	Transverse view to identify target (groove at root of TP and base of SAP)	Blocks performed at L1-L5; specific levels blocked are unclear; number of unilateral and bilateral cases not specified	None
Rauch^37^ 2009	Single-arm cohort	20	84	0.3 mL mixture of 1% lidocaine and steroid	Longitudinal view to determine level, transverse to determine target, lateral to medial in-plane injection	L3 MBB (28), L4 MBB (29), L5 MBB (35); number of unilateral and bilateral cases not specified	Fluoroscopic needle position
Shim^43^ 2006	Self-controlled cohort	20	101	1 mL 0.25% bupivacaine	Parasagittal view to determine level, transverse view to determine target (junction of cephalad TP and SAP), parasagittal view to confirm placement	T12 MBB (4), L1 MBB (22), L2 MBB (35), L4 MBB (31); number at L3 not reported but calculated to be 9 based on total number of blocks; number of unilateral and bilateral cases not specified	Fluoroscopic needle position and contrast
Soni^47^ 2018	Single-arm cohort	30	74	0.5 mL 2% lidocaine	US-guided MBB, technique not specified	Levels and laterality not specified	Fluoroscopic needle position and contrast (contrast not specifically mentioned in text but is noted on included confirmatory imaging)
Facet joint injection							
Constantinescu^9^ 2017	Case series	3	3 patients, total number of blocks not reported	Local anesthetic and steroid	Intra-articular placement verified by US, views not specified	Not specified	None
Erdogan^13^ 2019	Single-arm cohort	22	61	1 mL 2% lidocaine and 40 mg triamcinolone	Longitudinal view to determine level, transverse view with in-plane injection to superolateral corner of facet joint	Unilateral L3-4 (7), bilateral L3-4 (8), unilateral L4-5 (6), bilateral L4-5 (13), unilateral L5-S1 (4), bilateral L5-S1 (4); 6 levels could not be fully or partially visualized by US, although the specific levels were not specified	Fluoroscopic needle position and contrast
Galiano^16^ 2007	RCT	40 (US group: 20, CT group: 20)	20	1 mL 1% lidocaine, 1 mL 0.5% bupivacaine, and 4 mg betamethasone; 3 mL total volume	Parasagittal view to determine level, transverse view with in-plane injection to facet joint	L3-4 (1), L4-5 (6), L5-S1 (13); facet joints not able to be identified in 2 patients (level not specified), facets only partially identified in 2 other patients (level not specified)	CT needle position
Ha^19^ 2010	RCT	105 (US group: 54, control group: 51)	108	2% lidocaine and dexamethasone; 0.5 mL total volume	Parasagittal image to determine level, transverse view with in-plane injection	Bilateral L2-3 (3), bilateral L3-4 (15), bilateral L4-5 (28), bilateral L5-S1 (8)	None
Karkucak^25^ 2020	RCT	49 (US group: 25, palpation-guided: 24)	38	1% lidocaine and 10–20 mg triamcinolone per level; 1–2 mL total volume; 2nd injection performed at 2 wk	Parasagittal view to determine level, transverse view to determine target, lateral to medial in-plane injection	Unilateral L4-5 (18), unilateral L5-S1 (16), bilateral L5-S1 (2); 2 patients in US group did not complete the study	None
Kullmer^28^ 1997	Single-arm cohort	78	213	5 mL carbostesin in combination with corticosteroids	Transverse and longitudinal views to visualize facet joint; caudal to cranial in-plane injection	Bilateral L5-S1 (56), unilateral L5-S1 (2), unilateral L4-5 (1), bilateral L4-5 (46), bilateral L3-4 (3)	None
Sadeghian^40^ 2018	Case series	10	18	5 mg bupivacaine and 40 mg methylprednisolone	Longitudinal view to determine level, transverse view with in-plane injection	L4-5 and L5-S1, number of blocks per level not specified	None
Santiago^41^ 2014	Case report	3	3	0.25% bupivacaine and 10 mg methylprednisolone; 1 mL total volume	Longitudinal view to determine level, transverse view with out-of-plane injection	L1-2 (1), L2-3 (1), L3-4 (1)	Fluoroscopic needle position and contrast
Wen^51^ 2014	RCT	20 (US group: 10, CT group: 10)	37	0.5% lidocaine, 1–2 mL of analgesic solution	Facet joint identified with ultrasound in transverse plane, otherwise unspecified	Not specified	CT needle position
Ye^54^ 2018	RCT	40 (US group: 20, CT group: 20)	74	0.5 mL 2% lidocaine and 4 mg betamethasone; 2 mL total volume	Longitudinal view to determine level, transverse view to visualize facet joint	Not specified	CT needle position
Yun^55^ 2012	RCT	57 (US group: 25, control group: 32)	81	2 mL 1% lidocaine and 10 mg triamcinolone	Parasagittal view to identify level, transverse view with lateral to medial in-plane injection to midpoint of facet joint	Unilateral L4-5 (6), bilateral L4-5 (18), unilateral L5-S1 (5), bilateral L5-S1 (17)	None

BMI, body mass index; FL, fluoroscopic; FJI, facet joint injection; MBB, medial branch block; SAP, superior articular process; TP, transverse process; RCT, randomized controlled trial; US, ultrasound.

### 3.2. Risk of bias assessment

The full risk of bias assessment is presented in the supplemental materials document (available at http://links.lww.com/PR9/A160). Five RCTs were graded as having some concerns,^16,25,51,54,55^ while 1 was graded as high risk of bias because of bias in reporting outcomes.^19^ In the nonrandomized studies, 1 was graded as having good quality,^14^ 8 were graded as having fair quality,^3,13,21,24,28,37,40,43^ and 4 were graded as having poor quality.^9,17,32,47^ Significant risk of bias related to nonreporting of study data were identified, and most of the studies did not specify an a priori statistical plan.^3,9,13,17,19–21,24,25,32,37,40,43,47,55^ Three studies with high risk of bias because of nonreporting of information were conference abstracts.^9,32,47^ Some studies were susceptible to selection bias because of exclusion of patients with obesity.^25,54^ For all comparative studies, only 1 study reported that outcome assessors were blinded.^14^

### 3.3. Ultrasound-guided medial branch blocks

#### 3.3.1. Technique for performing ultrasound-guided medial branch blocks

The included studies describe T12-L5 MBB (L5 dorsal ramus blocks are herein referred to as MBB) with varying laterality and injectate volumes as detailed in Table 1. For US-guided MBB, 7 studies described a sagittal approach to identify the spinal level for injection followed by a transverse view to identify the target for final needle placement.^14,17,20,21,24,37,43^ One study only described using the transverse view.^32^ All studies which specified the target for needle placement described the junction of the cephalad transverse process and the superior articular process^14,17,20,21,24,32,43^ which has been shown in a cadaveric and CT-confirmation study as being less specific than targeting a lower point midway between the upper border of the transverse process and the mamilloaccessory ligament.^12^ Two studies did not describe the technique for performing the US-guided MBB.^3,47^ Six studies specified injections performed in-plane from a lateral to medial direction.^6,14,17,21,24,37^ One study described a reorientation of needle direction after performing an L4 MBB, in which the needle was withdrawn and walked medially and caudally while observing progress towards the target for the L5 MBB (intersection of sacral ala and superior articular process) in an out-of-plane fashion.^14^

Placement of 4 needles was associated with suspected vascular uptake because of contrast spread only partially covering target area in one study^14^ and because of lack of dye visualization under fluoroscopy in another.^43^ One study used a total volume of 0.25 mL, while the other used a total volume of 1 mL. The specific level of suspected intravascular uptake was not described. The number of patients with these suspected findings was not specified.

#### 3.3.2. Meta-analysis of ultrasound-guided medial branch blocks as confirmed by fluoroscopy

**Figure 2.**
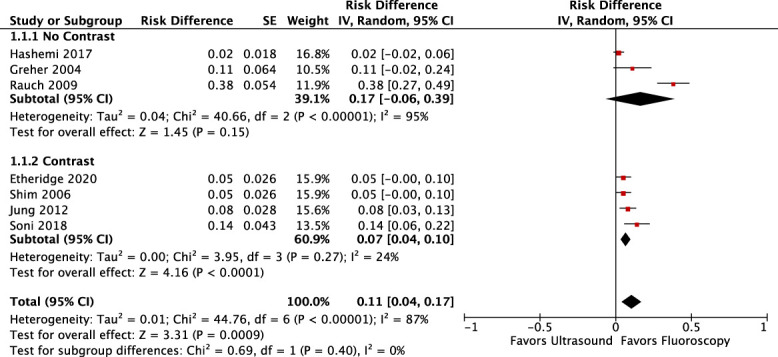
Risk difference forest plots for ultrasound-guided medial branch blocks confirmed by fluoroscopy with and without contrast.

Seven studies confirmed needle placement with fluoroscopy (Table 2).^14,17,21,24,37,43,47^ Three of 7 studies confirmed needle placement using fluoroscopy with contrast.^24,44,47^ Forest plots of the meta-analysis confirming correct needle placement using fluoroscopy with and without contrast are depicted in Figure 2. Pooled analysis demonstrated a 17% RD (95% CI, −0.06 to 0.39, *P* = 0.15) of incorrect needle placement for US-guided MBB confirmed using fluoroscopy without contrast with high levels of heterogeneity identified (*I*^*2*^ = 95%). Pooled analysis demonstrated a 7% RD (95% CI, 0.04–0.10, *P* < 0.0001) of incorrect needle placement for US-guided MBB confirmed using fluoroscopy with contrast with low levels of heterogeneity observed (*I*^*2*^ = 24%). Pooled analysis of all 7 studies demonstrated an 11% RD (95% CI, 0.04–0.17, *P* < 0.0009) of incorrect needle placement for US-guided MBB confirmed using fluoroscopy with and without contrast with high levels of heterogeneity identified (*I*^*2*^ = 87%). Heterogeneity was investigated by conducting a sensitivity analysis. When the study by Rauch et al.^37^ was removed from the meta-analysis, the RD in the fluoroscopy without contrast subgroup declined to 4% (95% CI, −0.03 to 0.12, *P* = 0.18) and statistical heterogeneity was reduced (*I*^*2*^ = 45%). The RD in the pooled analysis of the remaining 6 studies declined to 7% (95% CI, 0.04–0.10, *P* = 0.0002), and heterogeneity was reduced (*I*^*2*^ = 46%). The study cohort of Rauch et al.^37^ comprised exclusively of patients with a body mass index (BMI) greater than 30.

**Table 2:** Number of correct and incorrect needles placed by ultrasound for medial branch blocks and facet joint injections.

Author	Number of needles placed by US	Number confirmed as incorrect
US-guided MBB confirmed by fluoroscopy without contrast		
Greher^17^ 2004	28	3
Hashemi^21^ 2017	84	2
Rauch^37^ 2009	52	32
US-guided MBB confirmed by fluoroscopy with contrast		
Etheridge^14^ 2020^a^	100	5
Jung^24^ 2012	95	8
Shim^43^ 2006	101	5
Soni^47^ 2018	74	10
US-guided FJI confirmed by computerized tomography		
Galiano^16^ 2007	18	1
Wen^51^ 2014	42	5
Ye^54^ 2018	74	10
US-guided FJI confirmed by fluoroscopy with contrast		
Erdogan^13^ 2019	61	4

FJI, facet joint injection; MBB, medial branch block; US, ultrasound.

#### 3.3.3. Procedure time for a single-level ultrasound-guided medial branch blocks

Three studies reported the time needed to perform a single-level MBB.^21,37,43^ These studies reported that the average time ranged from 4.0 to 5.0 minutes.^21,37,43^ One of these studies also reported a total procedure time of 5.9 (SD 1) minutes, which may have included additional time to perform adjacent-level injections.^21^ Another study reported the time to perform L5 MBB in-plane and out-of-plane after reorientation of the needle from its position immediately after L4 MBB. Time for completion of this technique was reported as 153.93 (SD 41.56) seconds.^14^ An additional study reported that the procedure time for performing a US-guided MBB was significantly shorter compared with fluoroscopy (323 vs 430 seconds, *P* < 0.001).^20^ It was not clear from the methods of this study whether performance time was for a single-level or multilevel procedure. Another study reported that the maximum procedure time for multiple blocks at multiple levels was 40 minutes.^17^

#### 3.3.4. Complications associated with ultrasound-guided medial branch blocks

Complications were reported in 3 studies. Dizziness and bilateral lower extremity weakness were reported in 1 patient immediately after US-guided MBB.^32^ A vasovagal reaction was noted in 4 patients.^20^ Procedure level and laterality were not defined in these cases. Transient headache was noted in 2 patients.^20^ A small superficial hematoma was noted in 1 patient who underwent unilateral L4 and L5 MBB.^14^

### 3.4. Ultrasound-guided intra-articular facet joint injections

#### 3.4.1. Technique for performing ultrasound-guided facet joint injections

The included studies describe L1-2, L2-3, L3-4, L4-5, and L5-S1 FJI with varying laterality and injectate volumes as detailed in Table 1. Eight studies described a sagittal view to determine the spinal level followed by a transverse view to identify the target facet joint.^13,16,19,25,40,41,54,55^ One study described using both longitudinal and transverse views to identify the target with injection performed in-plane in a caudal to cranial trajectory.^28^ One study described confirmation of intra-articular injection with US but did not describe the particular views that were used.^9^ Eight studies described an in-plane approach,^13,16,19,25,28,40,54,55^ and 1 case report described an out-of-plane approach.^41^

#### 3.4.2. Ultrasound-guided facet joint injections confirmed by fluoroscopy with contrast

In a single study of US-guided FJI, correct needle position was confirmed using fluoroscopy with contrast (Table 2). A 7% RD was observed (95% CI, −0.00 to 0.13, *P* = 0.06).^13^

#### 3.4.3. Meta-analysis of ultrasound-guided facet joint injections confirmed by computerized tomography

**Figure 3.**
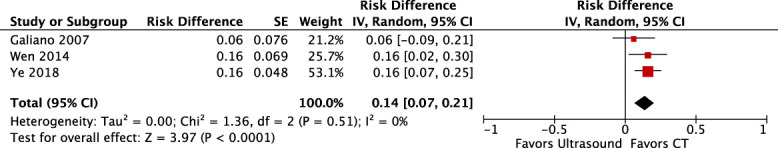
Risk difference forest plots for ultrasound-guided facet joint injections confirmed by computerized tomography.

Three studies confirmed US-guided needle placement with CT (Table 2). A forest plot of the meta-analysis confirming correct needle placement using CT is depicted in Figure 3. Pooled analysis demonstrated a 13% RD (95% CI, 0.06–0.19, *P* < 0.0001) of incorrect needle placement for US-guided FJI confirmed using CT, and no heterogeneity was identified (*I*^*2*^ = 0%).

#### 3.4.4. Procedure time for ultrasound-guided facet joint injections

Two studies reported the procedure time for performing a single intra-articular FJI at L3-4, L4-5, and L5-S1.^16,51^ One of these studies found a nonsignificant difference for the US-guided group (14.3 minutes, SD 6.6) compared with the CT-guided group (22.3 minutes, SD 6.3).^16^ Notably, in this study, the time recorded for the US-guided group also included the time expended obtaining CT control images.^16^ The other study reported a time of 206 seconds (SD 27 seconds) to perform a single-level FJI.^51^ Ha et al.^19^ measured the time to complete bilateral L2-3, L3-4, L4-5, and L5-S1 FJI, with no significant difference in procedure time observed between the US-guided (265 seconds) and fluoroscopy groups (247 seconds).^19^ Yun et al.^55^ measured time to complete multiple-level FJI.^55^ In this study, 25 patients underwent US-guided L4-5 and L5-S1 FJI for a total of 81 injections, while 32 patients underwent fluoroscopically guided L4-5 and L5-S1 FJI for a total of 104 injections. The procedure time in the US-guided group (263.4 seconds, SD 6.5) was significantly longer compared with the fluoroscopy group (248.7 seconds, SD 5.9, *P* = 0.023).^55^ In the study by Constantinescu et al.,^9^ which did not have a comparison group, the total US-guided procedure time ranged between 20 and 30 minutes. The number and levels of the injections were not reported.^9^ The definition and measurement of procedure time varied across studies.

#### 3.4.5. Complications associated with ultrasound-guided intra-articular facet joint injections

Complications were reported in 2 studies. Fluid retention in the upper and lower extremities was reported in 1 patient, although it was unclear whether this patient was in the US-guided or CT-guided group.^16^ Other details including level of the injection or time course of the symptoms were not reported. In the study by Ha et al.,^19^ a superficial infection that improved within a few days was reported. Whether antibiotics were administered was not reported. In the same study, an episode of transient lower motor neuron weakness that improved within 1 day was reported.^19^ The distribution of weakness was not reported. Several minor complications were reported in 4 patients in the US-guided FJI group and 3 patients in the fluoroscopically guided FJI group, but specific details about which complication occurred in each treatment group were not reported.^19^ These minor complications included aggravation of LBP, paresthesia, headache, brief chest pain, and an allergic reaction. All symptoms attributed to the minor complications resolved within a few hours.^19^

### 3.5. Grading of evidence

Certainty in evidence was assessed as low to very low primarily because of factors related to risk of bias, inconsistency, and imprecision.^18^ The complete assessment is presented in Table 3. Imprecision was primarily due to small sample sizes. Indirectness was noted because the image-guided interventions required highly specialized skills that may not be easily translated to health care personnel with less experience.^14,16,20,21,25,32,37,40,41,54,55^

**Table 3:** Grading of recommendations, assessment, development, and evaluation (GRADE) of evidence.

	Quality Assessment					Certainty in outcomes
	Risk of bias	Imprecision	Inconsistency	Indirectness	Publication bias	
Medial Branch Blocks						
Accuracy of injection	Moderate risk of bias primarily from selection bias	Imprecision because of relatively small sample sizes	Some inconsistency from lack of a priori statistics	Some concern of indirectness because of a specialized skill set required to perform the procedure that may not be widely available	Moderate risk given results that studies nearly universally favor US-guided MBB as feasible and many studies with only a single proceduralist	Low
Procedure time	Moderate risk of bias primarily from selection bias	Imprecision because of relatively small sample sizes	High inconsistency from lack of a priori statistics	Some concern of indirectness because of a specialized skill set required to perform the procedure that may not be widely available	Moderate risk given many studies with only a single proceduralist	Very low
Facet Joint Injections						
Accuracy of injection	Moderate risk of bias primarily from selection bias	Imprecision because of relatively small sample sizes	Some inconsistency from lack of a priori statistics	Some concern of indirectness because of a specialized skill set required to perform the procedure that may not be widely available	Moderate risk given results that nearly universally favor US-guided FJI as feasible and many studies with only a single proceduralist	Low
Procedure time	Moderate risk of bias primarily from selection bias	Imprecision because of relatively small sample sizes	High inconsistency from variability of effects and lack of a priori statistics	Some concern of indirectness because of a specialized skill set required to perform the procedure that may not be widely available	Moderate risk given many studies with only a single proceduralist	Very low

## 4. Discussion

The key findings of this systematic review include the following: (1) The pooled RD of US-guided MBB confirmed by fluoroscopy with or without contrast was 11%, and no significant group differences were observed; (2) the RD of US-guided FJI confirmed by fluoroscopy with contrast was 7%; and (3) the pooled RD of US-guided FJI confirmed by CT was 13%. The time to complete a single-level US-guided MBB ranged from 2.6 to 5.0 minutes, and a single study reported a significantly shorter procedure time for US-guided MBB compared with fluoroscopic guidance.^20^ However, the time to complete a single or multilevel US-guided FJI varied widely. Few complications were reported for US-guided, fluoroscopically guided, or CT-guided procedures. Important sources of heterogeneity and bias were identified, and the certainty in evidence was low to very low.

The RD of US-guided MMB and FJI as confirmed by fluoroscopy or CT warrants further consideration. Ultrasound technology is based on the piezoelectric principle, whereby electrical current passing through crystals in the US transducer are converted into pulsed sound waves.^1,30,53^ These ultrasonic waves are transmitted into the targeted tissues and reflected back to the transducer.^1,53^ High frequency transducers with shorter pulse length yield a higher resolution image. However, resolution is substantially limited when visualizing deeper structures because of attenuation of sound waves through the intervening tissues.^1,27,45,46^ Depth gain compensation can correct for the loss of acoustic energy through attenuation,^36,45^ but for deeper structures, depth gain compensation is inadequate for optimal visualization. Individual patient factors such as increased BMI and variations in adipose tissue distribution can contribute to suboptimal resolution.^10,34^ Thus, it can be posited that the technological limitations of US and individual patient factors are key contributors to the lower accuracy of US-guided MBB and FJI.

Despite the lower accuracy of US-guided needle placement, a previous meta-analysis reported the effectiveness of US-guided FJI were comparable with fluoroscopy-guided and CT-guided FJI.^52^ In this study, immediate postprocedural outcomes were assessed including pain scores, Modified Oswestry Disability (MOD) scores, and procedure time. Inclusion criteria included randomized and nonrandomized trials. The meta-analysis involved 2 fluoroscopy-guided trials^19,55^ and 1 CT-guided trial^16^; these 3 trials were included in our systematic review. In the meta-analysis, the weighted mean difference in pain scores, MOD scores, and procedure time did not differ significantly between the US-guided group and the combined fluoroscopy-guided and CT-guided group. However, high levels of statistical heterogeneity were identified for the pain score and procedure time analyses. No statistical heterogeneity was identified for the MOD analysis, but this comparison only included the 2 fluoroscopy trials.^19,55^ The findings of this systematic review and meta-analysis extend the findings of the meta-analysis of Wu et al.^52^ by including trials of US-guided MBB and reporting the RD of inaccurate needle placement. The findings of this review suggest that although the immediate postprocedural pain scores of US-guided FJI were similar to conventional imaging modalities, the risk of inaccurate needle placement could have deleterious effects on the diagnostic accuracy of MBB.

The findings of this systematic review have important implications for research and clinical practice. First, in a summary by Cohen et al.,^7^ the false-positive rate of fluoroscopically guided MBBs based on placebo-controlled blocks in randomized trials varied from 16% to 30%.^8,38,39,42^ The false-negative rate may be magnified by imaging modalities that miss the target nerve or cannot reliably detect intravascular uptake.^11^ The findings of this meta-analysis suggest that US-guided MBB could further impede the ability to accurately identify patients for radiofrequency denervation. However, use of US may be indicated in austere environments or select clinical scenarios where avoiding radiation exposure is a key outcome. The use of US may also be considered when diagnostic accuracy is a secondary concern. For example, as suggested by the findings of the meta-analysis of Wu et al.,^52^ the therapeutic effects of US-guided FJI may not be affected by inaccurate needle placement; thus, US may be an acceptable imaging modality for these injections. Further research using cadaver dissection models and prospective clinical trials are needed to drive development of techniques aimed at reducing the risk and understanding the clinical effects of incorrectly placed needles. Second, in the study by Rauch et al. that involved adults with a BMI >30 undergoing US-guided MBB, the RD was 38%. This finding is consistent with numerous studies where BMI >30 was associated with an increased risk of failed nerve blocks for regional anesthesia.^10,15,34,50^

This study has limitations. Details about how the US-guided procedures were performed varied between studies which could have influenced the findings of this systematic review. Training in fluoroscopically guided spine procedures is more extensive than US training. As a result, the outcomes of studies conducted by practitioners with expertise in performing US-guided procedures may not be generalizable to the general population of pain specialty physicians. Potential variations in how fluoroscopy was used without contrast to confirm needle placement could have affected the study findings. More specifically, no significant RD was observed for the US and fluoroscopy without contrast comparison (Fig. 2). The lack of significance could be due, in part, to high levels of heterogeneity which could be related to undefined variations in how fluoroscopy was used without contrast to confirm needle placement.

**Figure 4.**
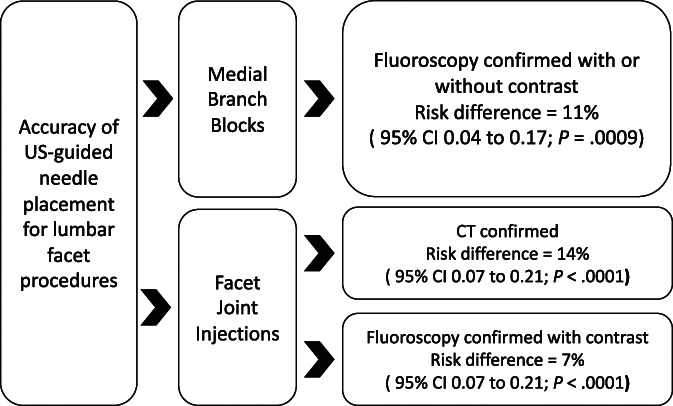
Summary of key study findings.

In conclusion, the risk of incorrect needle placement associated with US-guided MMB and FJI is high when needle position is confirmed using fluoroscopy or CT (Fig. 4). The technical limitations of US and individual patient characteristics, particularly elevated BMI, could be important determinants of incorrect needle placement associated with US-guided MBB and FJI. Further research is needed to identify optimal procedural techniques aimed at reducing the risk of incorrect needle placement for US-guided facet interventions.

## Disclosures

The authors have no conflicts of interest to declare.

## Appendix A. Supplemental digital content

Supplemental digital content associated with this article can be found online at http://links.lww.com/PR9/A160.

## Supplementary Material
