# Supplementary material for: Ultrasound-guided lumbar medial branch blocks and intra-articular facet joint injections: a systematic review and meta-analysis
Source: Pain Rep. 2022 May 16;7(3):e1008. doi: 10.1097/PR9.0000000000001008 (PMC9113209; doi:10.1097/PR9.0000000000001008)

**SUPPLEMENTAL MATERIALS**

**Search Strategy**

**Ovid**

Database(s): **Ovid MEDLINE(R) 1946 to Present and Epub Ahead of Print, In-Process & Other Non-Indexed Citations and Ovid MEDLINE(R) Daily, EBM Reviews - Cochrane Central Register of Controlled Trials**January 2020**, EBM Reviews - Cochrane Database of Systematic Reviews**2005 to February 27, 2021**, Embase**1974 to 2021 February 28
Search Strategy:

| **#** | **Searches** |
| --- | --- |
| 1 | exp Ultrasonography/ |
| 2 | (ultrasoun* or ultrason* or sonograph* or sonoanatom*).mp. |
| 3 | 1 or 2 |
| 4 | exp Nerve Block/ |
| 5 | (block or anesth* or anaesth*).mp. |
| 6 | "Anesthetics, Local"/ |
| 7 | or/4-6 |
| 8 | exp Back Pain/ |
| 9 | 3 and 7 and 8 |
| 10 | exp Cervical Vertebrae/dg, ir |
| 11 | exp Lumbar Vertebrae/dg, ir |
| 12 | exp Thoracic Vertebrae/dg, ir |
| 13 | ("medial branch" or facet* or zygapophysial or "dorsal ram*" or (low* adj back)).mp. |
| 14 | ((cervical or neck or lumbar or lumbosacr* or thoracic) adj1 (joint* or vertebra* or vertaebra* or spine or spinal)).ti. |
| 15 | ((c1 or c2 or c3 or c4 or c5 or c6 or c7) adj1 (joint* or vertebra* or vertaebra* or spine or spinal)).mp. |
| 16 | (thoracic adj1 (joint* or vertebra* or vertaebra* or spine or spinal)).mp. |
| 17 | ((t1 or t2 or t3 or t4 or t5 or t6 or t7 or t8 or t9 or t10 or t11 or t12) adj1 (joint* or vertebra* or vertaebra* or spine or spinal)).mp. |
| 18 | ((lumbar or lumbosacral or back) adj1 (joint* or vertebra* or vertaebra* or spine or spinal)).mp. |
| 19 | ((l1 or l2 or l3 or l4 or l5) adj1 (joint* or vertebra* or vertaebra* or spine or spinal)).mp. |
| 20 | or/10-19 |
| 21 | "pain management"/ |
| 22 | pain/ |
| 23 | pain.mp. |
| 24 | or/21-23 |
| 25 | 3 and 7 and 20 and 24 |
| 26 | ((ultrasoun* or ultrason* or sonograph* or sonoanatom* or image*) adj3 (interventional or needle* or visuali* or visibility or injection* or guid* or technique* or procedure*) adj7 (MBB or "medial branch" or block) adj7 (facet* or joint* or vertebra* or vertaebra* or spine or spinal or zygapophysial or "dorsal ram*" or (low* adj back))).mp. |
| 27 | 9 or 25 or 26 |
| 28 | limit 27 to english language [Limit not valid in CDSR; records were retained] |
| 29 | limit 28 to no language specified [Limit not valid in CDSR; records were retained] |
| 30 | 28 or 29 |
| 31 | 30 not ((exp animals/ or exp nonhuman/) not exp humans/) |
| 32 | remove duplicates from 31 |

**SCOPUS**

| 1 | TITLE ( ( ultrasoun* OR ultrason* OR sonograph* OR sonoanatom* ) ) |
| --- | --- |
| 2 | TITLE ( block ) |
| 3 | TITLE-ABS-KEY ( ( "medial branch" OR facet* OR zygapophysial OR "dorsal ram*" OR ( low* W/ back ) ) ) |
| 4 | TITLE-ABS-KEY ( ( ( cervical OR neck OR lumbar OR lumbosacr* OR thoracic ) W/1 ( joint* OR vertebra* OR vertaebra* OR spine OR spinal ) ) ) |
| 5 | TITLE ( ( ( c1 OR c2 OR c3 OR c4 OR c5 OR c6 OR c7 ) W/1 ( joint* OR vertebra* OR vertaebra* OR spine OR spinal ) ) ) |
| 6 | TITLE-ABS-KEY ( ( thoracic W/1 ( joint* OR vertebra* OR vertaebra* OR spine OR spinal ) ) ) |
| 7 | TITLE ( ( ( t1 OR t2 OR t3 OR t4 OR t5 OR t6 OR t7 OR t8 OR t9 OR t10 OR t11 OR t12 ) W/1 ( joint* OR vertebra* OR vertaebra* OR spine OR spinal ) ) ) |
| 8 | TITLE-ABS-KEY ( ( ( lumbar OR lumbosacral OR back ) W/1 ( joint* OR vertebra* OR vertaebra* OR spine OR spinal ) ) ) |
| 9 | TITLE-ABS-KEY ( ( ( l1 OR l2 OR l3 OR l4 OR l5 ) W/1 ( joint* OR vertebra* OR vertaebra* OR spine OR spinal ) ) ) |
| 10 | 3 or 4 or 5 or 6 or 7 or 8 or 9 |
| 11 | TITLE ( pain ) |
| 12 | 1 and 2 and 10 and 11 |
| 13 | TITLE ( ( ultrasoun* OR ultrason* OR sonograph* OR sonoanatom* OR image* ) AND ( interventional OR needle* OR visuali* OR visibility OR injection* OR guid* OR technique* OR procedure* ) AND ( mbb OR "medial branch" OR block ) AND ( facet* OR joint* OR vertebra* OR vertaebra* OR spine OR spinal OR zygapophysial OR "dorsal ram*" OR ( low* W/ back ) ) ) |
| 14 | 12 or 13 |
| 15 | INDEX(embase) OR INDEX(medline) OR PMID(0* OR 1* OR 2* OR 3* OR 4* OR 5* OR 6* OR 7* OR 8* OR 9*) |
| 16 | 14 not 15 |
| 17 | DOCTYPE(ed) OR DOCTYPE(bk) OR DOCTYPE(er) OR DOCTYPE(no) OR DOCTYPE(sh) OR DOCTYPE(ch) |
| 18 | 16 not 17 |
| 19 | LANGUAGE(english) |
| 20 | 18 and 19 |

Quality assessment of randomized controlled trials using the Cochrane Risk

of Bias Tool.


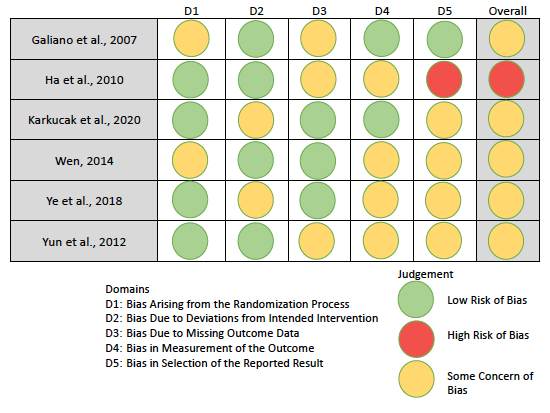


Quality assessment of cohort studies using the National Heart, Lung and Blood Institute tool.


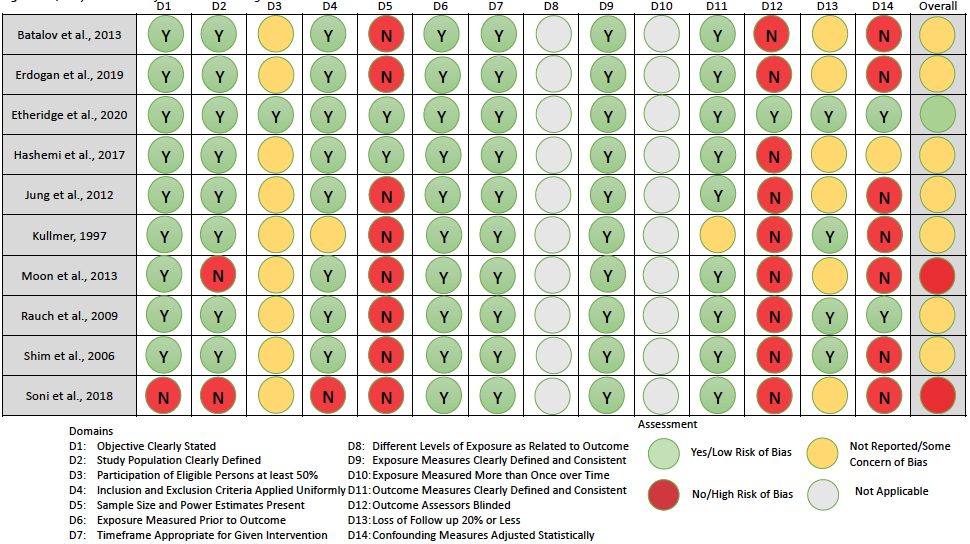


Quality assessment of case series studies using the National Heart, Lung and Blood Institute tool.


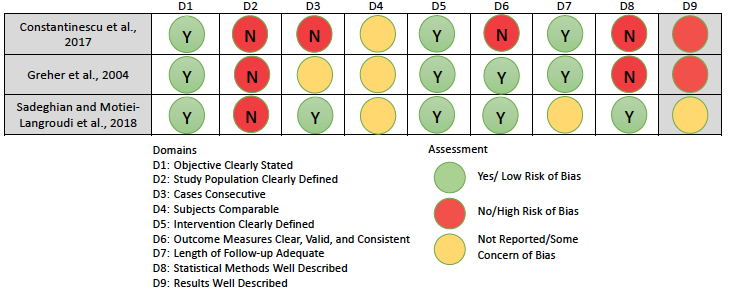

Supplement: Supplementary file 1 [file painreports-7-e1008-s001.docx]
